# Correlation analysis of the visual-analogue scale and the *Tinnitus Handicap Inventory* in tinnitus patients

**DOI:** 10.1016/S1808-8694(15)30835-1

**Published:** 2015-10-18

**Authors:** Ricardo Rodrigues Figueiredo, Andréia Aparecida de Azevedo, Patrícia de Mello Oliveira

**Affiliations:** 1 MSc in General Surgery, Otorhinolaryngology - Federal University of Rio de Janeiro, Assistant Professor of ENT - Medical School of Valença, RJ.; 2 MD. Otorhinolaryngologist - OTOSUL, Otorrinolaringologia Sul-Fluminense, Volta Redonda, RJ.; 3 Speech and Hearing Therapist, OTOSUL, Otorrinolaringologia Sul-Fluminense, Volta Redonda, RJ.

**Keywords:** measurement, tinnitus

## Abstract

One of the most challenging topics in tinnitus clinical studies is the measuring method used. Visual Analogue Scales (VAS) and Tinnitus Handicap Inventory (THI) are frequently used in tinnitus. **Aim:** To verify the relationship between VAS and THI scores in tinnitus patients in a prospective study. **Materials and methods:** 43 patients classified their tinnitus according to VAS and THI, and both scores were compared through the Spearman’s correlation coefficient test. **Results:** There was a correlation between the VAS and THI scores. **Conclusion:** There is correlation between VAS and THI scores in patients with sensorineural tinnitus.

## INTRODUCTION

Tinnitus is defined as the perception of a sound in the absence of an external sound source. Epidemiological studies report tinnitus incidence of 1 to 32% of the population, estimating between 35 and 50 million people in the USA. This incidence increases in the elderly population, reaching up to 15% in the age range above 65 years^1^, ^2^.

We may classify tinnitus in para-auditory (generated by muscular and vascular structures near the auditory pathways) and auditory (generated by alterations in the ear and auditory pathways). Among the latter, most cases correspond to the so-called “sensorineural tinnitus”^3^. The most recent theories consider tinnitus being generated at a cochlear level, with later perception in the central auditory pathways^4^, ^5^.

Tinnitus treatment is, until current days, one of the major challenges faced by otolaryngologists. Among the many factors responsible for this difficulty, one of them is, without doubt, how precarious are the tinnitus measurement and assessment methods, and there is no consensus in the literature as to the ideal assessment method.

**Figure 1:**
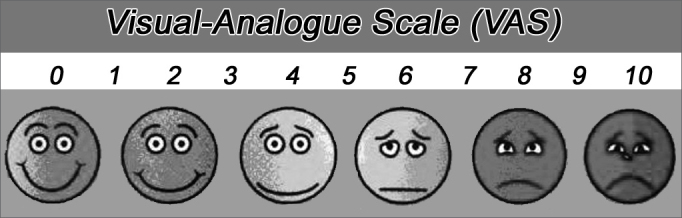
Model of the visual-analogue scale (VAS) used.

One of the most used method is the visual-analogue scale (VAS), very much used to assess chronic pain. In tinnitus patients, we ask the patient to assign a 0 to 10 score to their tinnitus, with the help of a proper ruler (Fig.1). The assessment must be carried out in relation to volume and disturbance. It is easily applicable and understood by most patients. However, this is a superficial assessment, impacted by cultural, intellectual and psychological aspects^6^.

In 1996, Newman et al. published a paper about the development of a Tinnitus Handicap Inventory (THI), through observing and studying other methods, such as Tinnitus Handicap/Support Questionnaire, Tinnitus Effect Questionnaire, Tinnitus Severity Questionnaire and Tinnitus Reaction Questionnaire. According to the authors, its main goal was to create a method with the following characteristics^7^:

- Summarized and proper for daily clinical practice
- Easy application and interpretation
- approaching many tinnitus aspects in the patient’s quality of life
- Validity and reliability.

Clinical data of patients with tinnitus and data from other scales were used in order to develop THI. Three main items are assessed in THI, namely^7^:

- Functional reactions to tinnitus, such as difficulties to concentrate and anti-social trends;
- Emotional reactions to tinnitus, such as anger, frustration, irritability, depression;
- catastrophic reactions to tinnitus, such as despair, a feeling of hopelessness, a fear of a “severe disease”, loss of control and incapacity to cooperate.

**Chart 1:**
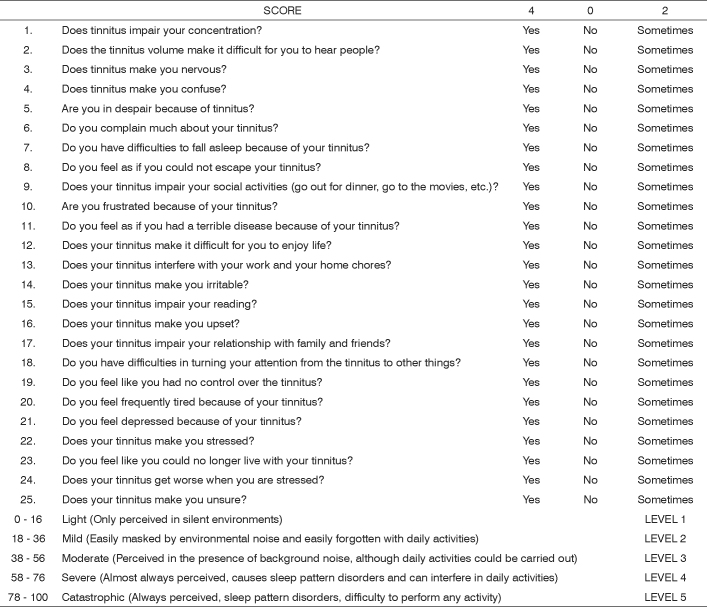
THI questionnaire adapted to Brazilian Portuguese (Ferreira PEA, Cunha F, Onishi ET, Branco FCA, Ganança FF).

THI is today one of the most accepted method to assess tinnitus, having been advocated in many consensus. THI validation to Brazilian Portuguese (Chart 1) was carried out in 2005^8^.

Our objective in the present study is to assess the correlation between VAS scores and THI in patients with sensorineural hearing loss.

## MATERIALS AND METHODS

We selected 43 patients with tinnitus seen in our service between March 2006 and January 2007. Inclusion criteria were sensorineural-related tinnitus, and we ruled out cases of concurrent external and middle ear diseases and TMJ disorders. Tonal and vocal audiometry and impedance tests were carried out in all the patients, and we took off those with conductive hearing loss, mixed hearing loss and those with types A-r, A-d, C and B tympanic curves. The audiometer we used was an AMPLAID A 177 PLUS, and the AMPLAID 750 impedance meter.

We asked the patients to fill out a validated questionnaire, in the case of THI (Tinnitus Handicap Inventory) in its Portuguese version. Moreover, the patients classified their tinnitus according to the visual-analogue scale, from 1 to 10 (in terms of volume and disturbance), and we correlated THI and VAS scores through the Spearman’s coefficient correlation. Spearman’s correlation coefficient (rs) measures the level of association between two variables. This coefficient varies from -1 to 1, the closer it is to 1 or -1, the stronger is the association the closer it is to zero, the weaker the relation between the two variables. The negative coefficient expresses an inverse relationship between the two variables.

The study was approved by the Ethics in Research Committee of the Valença Medical School, under protocol # 003/2006.

## RESULTS

**Chart 2:**
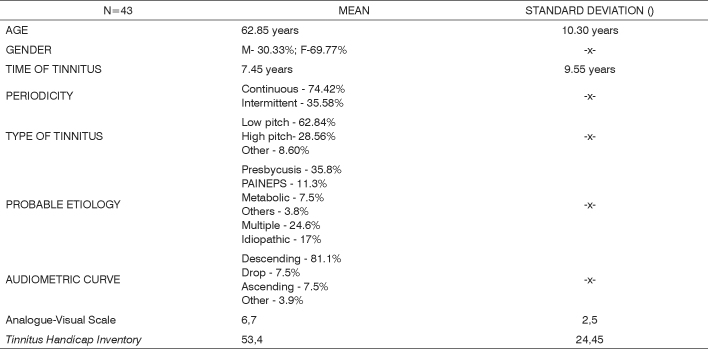
Sample characterization.

The data on the sample characterization can be found on Chart 2.

**Figure 2:**
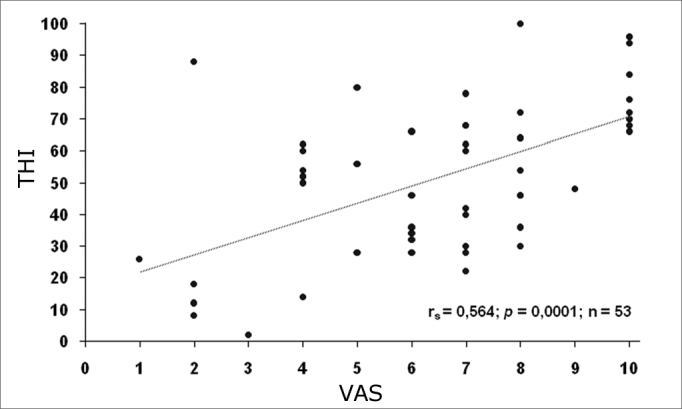
Relationship between VAS and THI scores.

According to the Spearman’s relation coefficient, we observed that there is a significant correlation between THI and VAS (rs = 0.564; p = 0.0001; n = 43). This means that, the higher the VAS, the higher the expected value for THI (direct relation), as shown on Fig.2.

## DISCUSSION

A large part of the numerous criticisms regarding tinnitus clinical trials are associated with the method of assessment. In searching for the ideal measurement method, many questionnaires have been proposed, and the THI is one of the most employed in the international literature. In Brazil, most clinical studies were already carried out used VAS; however, the most recent studies carried out have been done so with THI.

The correlation between the two methods, seen in our data, increases the reliability of the studies already carried out with VAS, a simpler method and, in our opinion, it is easier to understand by the majority of the Brazilian population. However, we consider THI a more complete method for tinnitus assessment, especially when we consider the daily and psychological aspects of tinnitus.

Considering such facts, we started to employ both methods together in our clinical studies, and this makes our results more reliable, as we see it.

## CONCLUSION

There is a correlation between the Visual-Analogue Scale and the Tinnitus Handicap Inventory in patients with sensorineural tinnitus.
